# Genetic diversity of porcine reproductive and respiratory syndrome virus and evaluation of three one-step real-time RT-PCR assays in Korea

**DOI:** 10.1186/s12917-022-03407-0

**Published:** 2022-08-30

**Authors:** Go-Eun Shin, Ji-Young Park, Kyoung-Ki Lee, Mi-Kyeong Ko, Bok-Kyung Ku, Choi-Kyu Park, Hye-Young Jeoung

**Affiliations:** 1 Animal Disease Diagnostic Division, Animal and Plant Quarantine Agency, Gimcheon, 39660 Korea; 2 College of Veterinary Medicine, Kyungbuk National University, 80, Daehak-ro, Daegu, 41566 Korea

**Keywords:** Porcine reproductive and respiratory syndrome virus (PRRSV), Genetic diversity, Evaluation, Diagnostic method

## Abstract

**Background:**

Porcine reproductive and respiratory syndrome virus (PRRSV) has caused huge economic losses in the global swine industry. Frequent genetic variations in this virus cause difficulties in controlling and accurately diagnosing PRRSV.

**Methods:**

In this study, we investigated the genetic characteristics of PRRSV-1 and PRRSV-2 circulating in Korea from January 2018 to September 2021 and evaluated three one-step real-time reverse transcription polymerase chain reaction (RT-PCR) assays.

**Results:**

A total of 129 lung samples were collected, consisting of 47 samples for PRRSV-1, 62 samples for PRRSV-2, and 20 PRRSV-negative samples. Nucleotide sequence analysis of open reading frames (ORFs) 5, ORF6, and ORF7 genes from PRRSV samples showed that PRRSV-1 belonged to subgroup A (43/47, 91.49%) and subgroup C (4/47, 8.51%), whereas PRRSV-2 was classified as lineage 1 (25/62, 40.32%), Korean lineage (Kor) C (13/62, 20.97%), Kor B (10/62, 16.13%), lineage 5 (9/62, 14.52%), and Kor A (5/62, 8.06%). Amino acid sequence analysis showed that the neutralizing epitope and T cell epitope of PRRSV-1, and the decoy epitope region and hypervariable regions of PRRSV-2 had evolved under positive selection pressure. In particular, the key amino acid substitutions were found at positions 102 and 104 of glycoprotein 5 (GP5) in some PRRSV-2, and at positions 10 and 70 of membrane protein (M) in most PRRSV-2. In addition, one-step real-time RT-PCR assays, comprising two commercial tests and one test recommended by the World Organization for Animal Health (OIE), were evaluated.

**Conclusion:**

The results revealed that two of the real-time RT-PCR assays had high sensitivities and specificities, whereas the real-time RT-PCR assay of the OIE had low sensitivity due to mismatches between nucleotides of Korean PRRSVs and forward primers. In this study, we genetically characterized recent PRRSV occurrences and evaluated three one-step real-time RT-PCR assays used in Korea.

**Supplementary Information:**

The online version contains supplementary material available at 10.1186/s12917-022-03407-0.

## Background

Porcine reproductive and respiratory syndrome virus (PRRSV) is an enveloped, single-stranded positive-sense RNA virus belonging to the family *Arteriviridae* of the order *Nidovirales*. PRRSV causes reproductive failure in sows and respiratory distress in pigs of all ages, resulting in significant economic losses for the swine industry worldwide [1, 2]. The PRRSV genome contains 10 open reading frames (ORFs), including ORF1a, 1b, 2a, 2b, 3, 4, 5a, 5, 6, and 7 [3, 4]. ORF1a and ORF1b code for two large polyproteins that are generate 14 nonstructural proteins [4]. Eight structural genes (ORF2a, ORF2b, ORF3–7, and ORF5a) encode structural proteins, including glycoprotein (GP) 2, small envelope (E), GP3, GP4, GP5, membrane (M), nucleocapsid (N), and ORF5a proteins, respectively [5, 6]. PRRSV can be divided into two genotypes: European PRRSV type 1 (PRRSV-1) and North American PRRSV type 2 (PRRSV-2). Recently, PRRSV-1 was taxonomically classified into the species *Betaarterivirus suid 1* and PRRSV-2 into the species *Betaarterivirus suid 2* based on international committee on taxonomy of viruses (ICTV). The two prototype genomes, the Lelystad strain for PRRSV-1 and VR-2332 strain for PRRSV-2, share approximately 60% homology in their nucleotide sequences [7]. In Korea, PRRSV-2 has spread rapidly since its first report in 1994 [8], and PRRSV-1 was identified in 2005 [9]. Genetic diversity and phylogeny have been reported based on genetic analysis of ORF5 sequences of PRRSVs prevailing in Korea [10–13]. The Korean PRRSV-1 isolates belong only to subtype 1, whereas Korean PRRSV-2 isolates are classified as lineages 1, 4, 5, and Korean lineages (Kor) A, B, and C [11, 13, 14].

The ORF5 sequence of PRRSV has been widely used to study phylogeny, genetic variation, and molecular epidemiology [15]. Many PRRSVs that were genetically and geographically differentiated, were classified into subtypes 1–4 from PRRSV-1 and lineages 1–9 from PRRSV-2 [3, 16]. ORF6 encodes the most conserved structural protein of PRRSV. The phylogenetic tree derived from ORF7 resembles the tree derived from the full-length genomes of PRRSV [17]. The conserved regions of ORF6 and ORF7 are often used as target regions for PRRSV detection by nucleotide-based assays [18–22].

ORF5 encodes a highly variable envelope protein, GP5, which plays an important role in viral infectivity and contains immunological domains related to viral neutralization [23, 24]. GP5 and M protein, two major envelope proteins, form a disulfide-linked heterodimer or a disulfide-linked multimer that is essential for virion formation [25, 26]. GP4, GP5, and M proteins induce neutralizing murine monoclonal antibodies (MAbs). In particular, MAbs recognizing GP5 neutralize PRRSV more effectively than other MAbs [27]. Therefore, GP5 has been considered a major target protein for vaccine design as it is involved in the production of neutralizing antibodies, followed by protection against PRRSV [28]. The non-neutralizing epitope of PRRSV is highly immunodominant and exhibits some features of decoy epitopes, which have been demonstrated to inhibit recognition of neutralizing epitopes in several viral infections [28].

Nucleic acid-based diagnostic methods have been commonly used to diagnose PRRSV owing to their sensitivity, specificity, and relatively rapid test times [29–31]. However, RNA viruses, such as PRRSV and swine influenza virus (SIV), have high mutation rates, rapid evolution, and genetic variability, these complicate the development of reliable diagnostic methods [32–35]. Many studies have shown that genetic differences or mismatches between nucleotides of PRRSV and the primers in molecular-based assays can lead to false results [36–39]. Therefore, the continuously increasing genetic diversity of PRRSV with the emergence of new strains dictates the need for an accurate diagnosis.

In this study, we investigated the genetic diversity of PRRSVs circulating in Korea through phylogenetic analysis and amino acid analysis from January 2018 to September 2021 and evaluated three one-step real-time reverse transcription polymerase chain reaction (RT-PCR) assays used in Korea.

## Methods

### Clinical sample and detection of PRRSV

A total of 129 lung samples submitted to the Diagnostic Division of the Animal and Plant Quarantine Agency (APQA) to diagnose swine disease, were collected from January 2018 to September 2021. The samples were collected from farms across all Korean provinces and included mainly clinical signs of PRRS such as acute respiratory disease in growing pigs or late-term abortion in sows. All lung tissue samples were homogenized with alpha modification of Eagle's minimum essential medium (EMEM) (Gibco, Grand Island, NY, USA) containing 1% antibiotic (Gibco). Viral RNA was extracted from the supernatant of tissue homogenates using the RNeasy Mini Kit (QIAGEN, Hilden, Germany) according to the manufacturer's instructions. Commercial VDX® PRRSV HP MP RT-PCR and NA/EU Typing Nested PCR (Median Diagnostics, Gangwon, South Korea) were used for detecting and genotyping PRRSV.

### Sequencing and phylogenetic analysis of ORF5, ORF6, and ORF7 of PRRSV

ORF5, ORF6, and ORF7 were amplified from PRRSV-positive samples by RT-PCR with specific primer sets (Table 1). RT-PCR amplification was performed under the following conditions: reverse transcription for 30 min at 50 °C, and termination of reverse transcription for 15 min at 95 °C, followed by 35 cycles of denaturation, annealing, and extension for 30 s at 94 °C, 30 s at 55 °C, 50 s at 72 °C, respectively, and a final extension of 10 min at 72 °C. The PCR products were sequenced by commercial sequencing service company (Macrogen, Daejeon, South Korea). Some ORF5, ORF6, and ORF7 genome sequences among the all sequences obtained in this study were submitted to GenBank under accession number ON892744–ON892781. Reference strains, such as global PRRSV strains, Korean PRRSV strains, and two PRRSV prototype strains (Lelystad and VR2332) obtained from National Center for Biotechnology Information (NCBI), were included in the dataset for phylogenetic analysis. A phylogenetic tree was generated by maximum likelihood analysis using the Kimura two-parameter model (K2P) with MEGA 7.0 (Pennsylvania State University, State College, Pennsylvania, USA), and was evaluated by 1,000 bootstrap replicates. The Markov Chain Monte Carlo (MCMC) algorithms implemented in the BEAST v1.7.5 package was used to estimate the substitution rate per site per years (s/s/y) of the Korean PRRSV strain from 1997 to 2021. The dataset consisted of a total of 238 PRRSV-1 ORF5 sequences and 319 PRRSV-2 ORF5 sequences including Korean PRRSV reference strains available in NCBI and ORF5 sequences obtained in this study. Evolutionary rate was estimated using the relaxed molecular clock model with GTR + Γ4 mixed substitution according to a previous study [40].

**Table 1:** Primers for RT-PCR amplification of ORF 5, ORF6, and ORF7 genes of PRRSV

Genotype	Target	Primer	Sequence 5' – 3'	Reference
PRRSV-1	ORF5	EU ORF5 F	CCGTCTGTGATGAGRTGGGC	Kang et al., 2018 [13]
		EU ORF5 R	GGAYACTTTTAGGGCRTATA	
	ORF6	EU ORF6 F	GTCGTCCTCGAAGGGGTTAAAG	In this study^a^
		EU ORF6 R	YGGCGCTGGGACTTYATCA	
	ORF7	EU ORF7 F	GCATACGCTGTGAGAAAGC	
		EU ORF7 R	CTATTCAATTAGGGCGACCGTG	
PRRSV-2	ORF5	NA ORF5 F	GTGGGCRACYGTTTTAGCCT	
		NA ORF5 R	CATAGTGAGCGCGACCYTAT	
	ORF6	NA ORF6 F	TYGTGCTTGATGGTTCCGYG	
		NA ORF6 R	AGYTGATTGACTGGCTGGCC	
	ORF7	NA ORF7 F	AACGGYACAYTGGTGCCC	
		NA ORF7 R	CTATTCAATTAGGGCGACCGTG	

^a^The target regions of Korean PRRSV-1 and PRRSV-2 strains collected from the NCBI database were aligned and the primer were designed in conserved sequences of target genes

### Amino acid analysis of GP5 and M protein

Amino acid analysis between lineages and sequence entropy at each codon indicating amino acid diversity was conducted according to a previous study [41]. Briefly, graphical sequence logos for each lineage were generated using the WebLogo tool (http://weblogo.berkeley.edu/) and sequence entropy was generated using the Shannon Entropy-One tool implemented in the HIV database tool (https://www.hiv.lanl.gov/). To determine the action of selection pressure on the structural proteins of PRRSV-1 and PRRSV-2, site-by-site selection at single codon sites of each structural protein was estimated using the mixed-effects maximum likelihood model of evolution available at DataMonkey (http://www.datamonkey.org/) [42, 43]. Sites with a *p*-value ≤ 0.05 were inferred to be positively selected.

### One-step real-time RT-PCR assays

Two commercially available certified one-step real-time RT-PCR assays (A and B tests) are the most commonly used for detecting PRRSV in Korea. Test A and B were performed using each kit provided under the same lot number. The one-step real-time RT-PCR (C test) is recommended in the OIE manual of diagnostic tests [44] and is used by a private animal disease diagnostic center. The C test was performed using the QuantiNova Probe RT-PCR kit (QIAGEN). The reaction mix was prepared using 10 μL of 2 × probe RT-PCR Master Mix, 0.2 μL of QN Probe RT-Mix, 3 μL of the final concentration of forward/reverse primer and probe mix according to a previously described protocol [45], 2 μL of RNase-free water, and 5 μL of RNA. Reactions were performed according to the manufacturer's instructions.

### Sensitivities and specificities of each assay using reference strains

The sensitivities of the assays were evaluated using two reference strains, the Lelystad strain for PRRSV-1 and LMY strain (GenBank No. DQ473474) for PRRSV-2. Lelystad virus (10^5.0^ 50% tissue culture infective dose per ml [TCID_50_/ml]) and LMY virus (10^5.2^ TCID_50_/ml) were serially tenfold diluted from 10^5^ to 10^–1^ TCID_50_/ml and applied to each one-step real-time RT-PCR. To estimate diagnostic specificity, other respiratory viruses, such as porcine circovirus type 2 and type 3 (PCV2 and PCV3), classical swine fever virus (CSFV), porcine parvovirus (PPV), and SIV were tested for cross-reactivity.

### Clinical evaluation of three one-step real-time RT-PCR assays

The three one-step real-time RT-PCR assays were evaluated for 129 clinical samples, and PRRSV-positive samples were determined by sequencing. In order to eliminate PCR inhibition of total RNA extracted from tissue samples, false-negative samples were diluted tenfold in five concentrations (10^–1^, 10^–2^, 10^–3^, 10^–4^, and 10^–5^) [46, 47]. The threshold value was set at 200 relative fluorescence units (RFU) above the noise band. The samples were tested in independent runs in separate rooms for a one-step real-time RT-PCR assay.

## Results

### Phylogenetic analysis of PRRSV

**Fig. 1:**
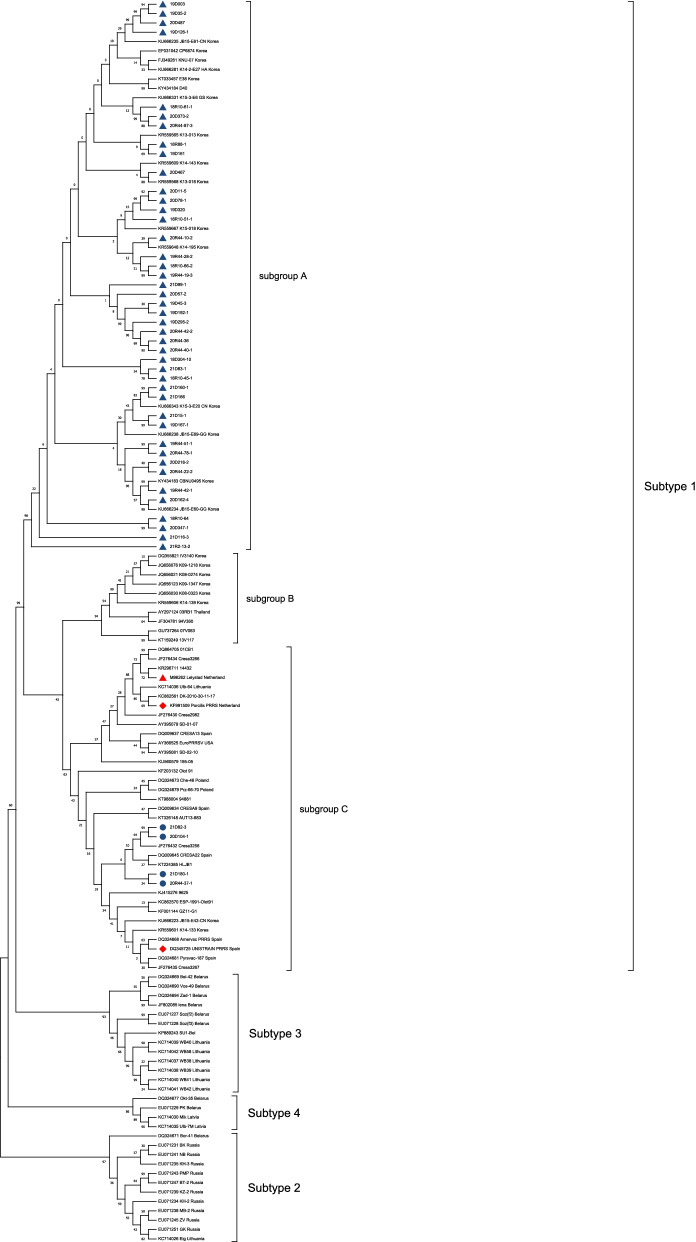
Phylogenetic analysis of ORF5 of PRRSV-1 isolates. The tree was constructed by maximum likelihood method. Prototype viruses, vaccine viruses used in Korean swine farms were marked with  and  , respectively. Subgroup A and C of field strains obtained from this study was marked with  and  , respectively

**Fig. 2:**
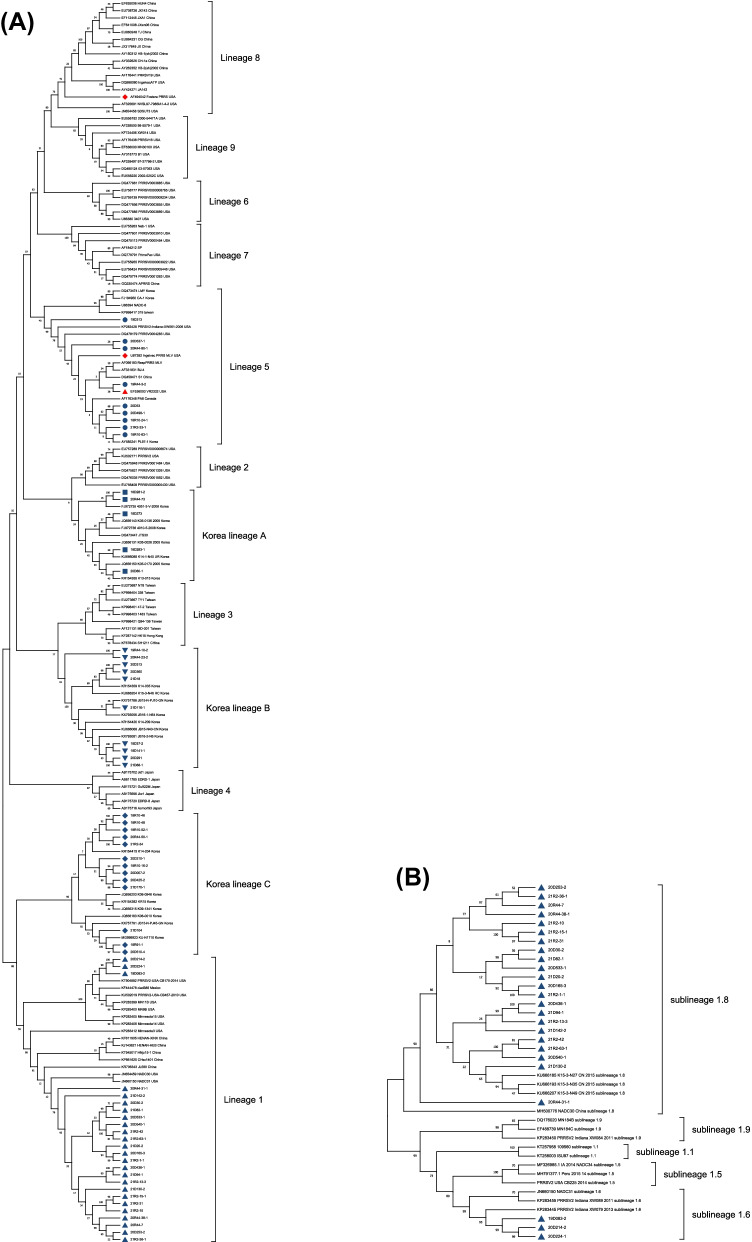
Phylogenetic analysis of ORF5 of PRRSV-2 isolates (**A**). The tree was constructed by maximum likelihood method. Prototype viruses, vaccine viruses used in Korean swine farms were marked with  and  , respectively. Lineage 1, lineage 5, Kor A, Kor B, and Kor C of field strains obtained from this study was marked with  ,  ,  ,  , and  , respectively. Genotyping of PRRSV-2 lineage 1 circulating in Korea (**B**)

PRRSV was detected in 109 samples, which consisted of 47 samples for PRRSV-1 and 62 samples for PRRSV-2. Twenty samples were negative for PRRSV. Through ORF5 analysis, the PRRSV-1 samples belonged to pan-European subtype 1 and were classified into subgroup A (43/47, 91.49%) and subgroup C (4/47, 8.51%) (Fig. 1). The PRRSV-1 nucleotide sequence homologies of ORF5 to Lelystad were 79.74–87.23% and 90.69–94.78% for subgroup A and subgroup C, respectively. The nucleotide sequence homologies of ORF5, ORF6, and ORF7 among the different PRRSV-1 samples were 79.59–99.83%, 85.85–100%, and 86.52–100%, respectively. The PRRSV-2 samples by ORF5 analysis were classified into lineage 1 (25/62, 40.32%), Kor C (13/62, 20.97%), Kor B (10/62, 16.13%), lineage 5 (9/62, 14.52%), and Kor A (5/62, 8.06%) (Fig. 2A). The PRRSV-2 nucleotide sequence homologies of ORF5 to VR2332 were 79.88–82.52%, 78.71–83.90%, 84.63–85.77%, 95.54–99.33%, and 82.78–86.14% for lineage 1, Kor C and B, lineage 5, and Kor A, respectively. The nucleotide sequence homologies of ORF5, ORF6, and ORF7 among different samples of PRRSV-2 were 74.81–100%, 83.21–99.81%, and 79.66–100%, respectively. Among lineage 1, 22 samples (22/62, 35.48%) could be classified into sublineage 1.8 (NADC30-like) and three samples (3/62, 4.84%) belonged to sublineage 1.6 (Fig. 2B). The mean evolutionary rates of 238 Korean PRRSV-1 and 319 PRRSV-2 were 6.931 × 10^–3^/site/year (95% HPD intervals from 5.8521 × 10^–3^ to 7.9748 × 10^–3^) and 5.131 × 10^–3^/site/year (95% HPD intervals from 4.516 × 10^–3^ to 5.7664 × 10^–3^), respectively.

### Amino acid analysis of GP5 and M protein from PRRSV-1

**Fig. 3:**
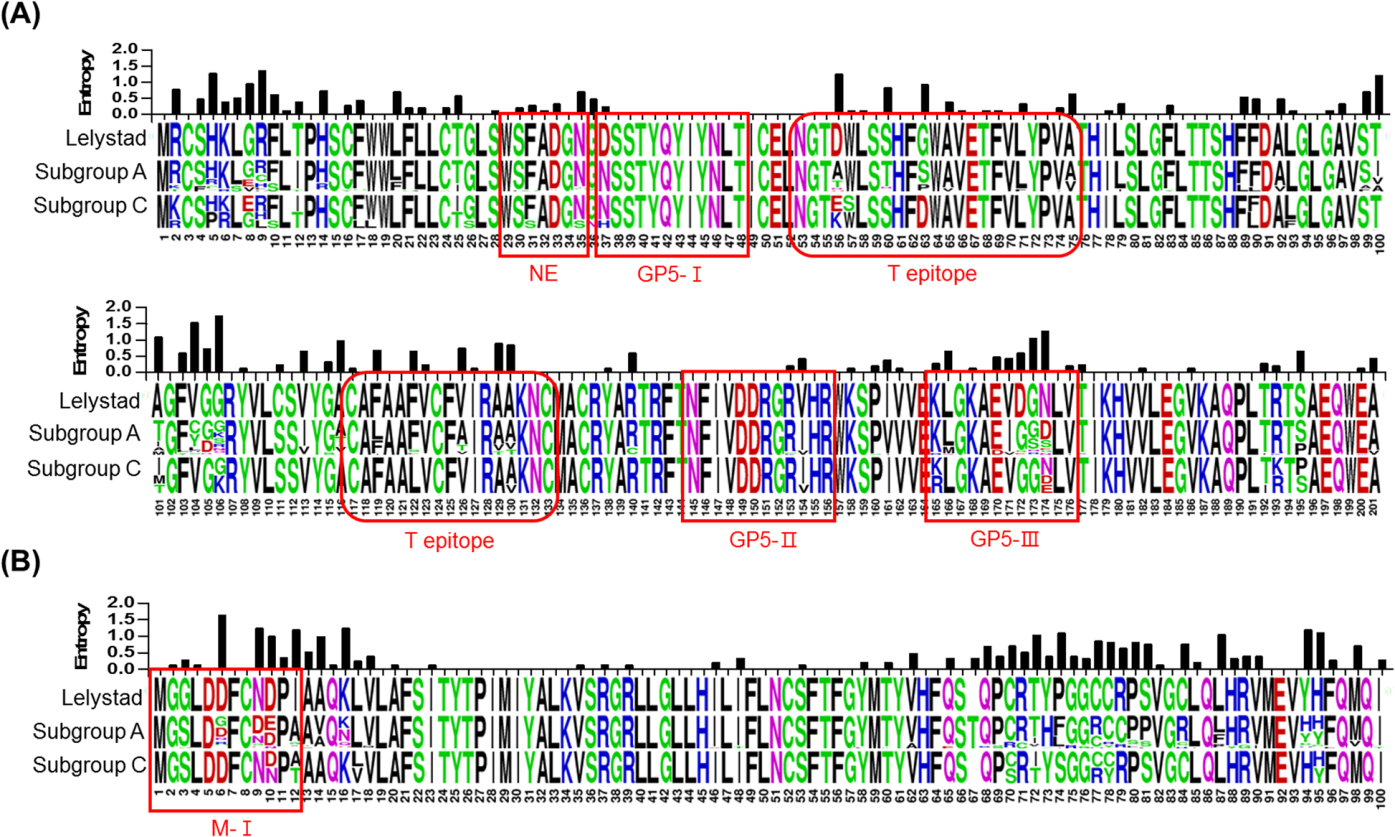
The alignment and entropy plot (amino acid diversity) of GP5 and M of PRRSV-1 samples with Lelystad strain. Multiple alignments of GP5 (**A**) and M (**B**) protein of PRRSV-1 are numbered from start of the GP5 domain (aa 1–201) and M protein domain (aa 1–100), respectively. The sequence of Lelystad is shown at the top of (**A**) and (**B**). The size of each aa letter at each sequence logos is proportional to the frequency. Amino acids are color-coded: blank, nonpolar; green, polar uncharged; red, polar with a positive charge; blue, polar with negative charge. A red square box indicates a B-cell epitope region, and a red round box indicates a T-cell epitope region. NE: neutralizing epitope, T epitope: T-cell epitope

Previous studies identified one neutralizing epitope at amino acid (aa) 29–35 in GP5, which was reported to be ^29^WSFADGN^35^ in the Lelystad strain. Additionally, there were T cell epitopes and four B cell epitopes (GP5-I, GP5-II, GP5-III, and M-I) in GP5 and M protein of PRRSV-1 [48–51]. As shown in Fig. 3, the neutralizing epitope was conserved with a low level of entropy. Subgroup A did not show much variation in the neutralizing epitope region, while only the 20R44-37–1 sample belonging to subgroup C showed variation in position 31 (^31^F → ^31^S) and 35 (^35^ N → ^35^S). By contrast, the GP5-III and M-I regions were variable with a high level of entropy, indicating genetic diversity (Fig. 3). A total of 16 codon sites were positively selected in the GP5 and M proteins of PRRSV-1 (Table 2). Interestingly, one positive selection site at position 35 was included in the neutralizing epitope region (positions 29–35), and two positive selection sites at positions 56 and 60 were included in the T cell epitope region (positions 53–75). Positive selection sites at positions 5, 7, 9, 20, 35, 36, and 104 in GP5 were observed with *p*-value < 0.01.

**Table 2:** Positive selection sites of Korean PRRSV identified between January 2018 and September 2021

Type	PRRSV-1				PRRSV-2			
Structural protein	GP5		M		GP5		M	
Positively selected sites	Site^a^	*P*-value	Site^a^	*P*-value	Site^b^	*P*-value	Site^b^	*P*-value
	2	0.03	58	0.02	2	0.03	10	< 0.01
	5	< 0.01			5	0.02	15	0.03
	7	< 0.01			6	0.03	16	< 0.01
	8	0.02			15	0.04	66	< 0.01
	9	< 0.01			26	0.03		
	12	0.04			28	0.01		
	14	0.03			30	0.04		
	20	< 0.01			32	< 0.01		
	22	0.04			33	0.01		
	35	< 0.01			34	< 0.01		
	36	< 0.01			35	0.02		
	56	0.02			59	0.04		
	60	0.01			61	0.03		
	103	0.03			73	0.02		
	104	< 0.01			98	0.02		
					102	0.05		
					104	0.05		
					151	0.05		

^a^Amino acid position based on Lelystad strain

^b^Amino acid position based on VR2332 strain

### Amino acid analysis of GP5 and M protein from PRRSV-2

**Fig. 4:**
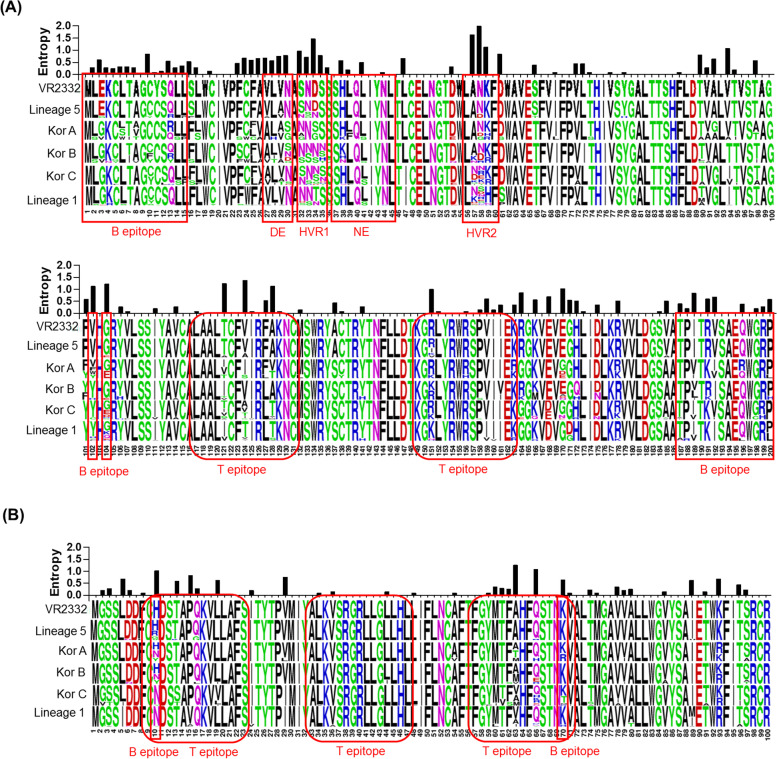
The alignment and entropy plot (amino acid diversity) of GP5 and M of PRRSV-2 samples with VR2332 strain. Multiple alignments of GP5 (**A**) and M (**B**) protein of PRRSV-2 are numbered from start of the GP5 domain (aa 1–200) and M protein domain (aa 1–100), respectively. The sequence of VR2332 is shown at the top of (**A**) and (**B**). The meaning of the size and color of the sequence logos is explained in the legend to Fig. 3. A red square box indicates a B-cell epitope region, and a red round box indicates a T-cell epitope region. DE: decoy epitope, NE: neutralizing epitope, HVR: hypervariable region, B epitope: B-cell epitope, T epitope: T-cell epitope

Both a decoy epitope and a neutralizing epitope located at GP5 of PRRSV-2 have been identified, comprising residues 27–30 (^27^VLVN^30^) and residues 37–45 (^37^SHLQLIYNL^45^), respectively (Ostrowski et al., 2002). The diversity of amino acid sequences occurred in two previously identified B cell epitopes, aa 1–15 and aa 187–200, and two previously identified T cell epitopes, aa 117–131 and aa 149–163 (de Lima et al., 2006; Vashisht et al., 2008; Zhou et al., 2009). As shown in Fig. 4A, critical amino acid variations in the B cell and T cell epitopes were also found in GP5 of PRRSV-2. In the decoy epitope compared with the VR2332 strain, significant diversity was found with higher amino acid entropy. Interestingly, a specific substitution at position 44 (^44^ N → ^44^ K) was found in the 21R2-15–1 sample belonging to lineage 1. In addition, 18R10-16–2 and 20D007-2 samples belonging to Kor C and 21D82-1 sample belonging to lineage 1 had a cysteine at position 102 in GP5. The 18R10-16–2 sample belonging to Kor C and 20D253-2 sample belonging to lineage 1 have an arginine at position 104 in GP5. Previous studies identified two B cell epitope regions at positions 10 and 70, and three T cell epitope regions at aa 9–23, aa 33–47, and aa 57–71 of M proteins [52–54]. The M protein region was relatively more conserved compared with that of GP5 (Fig. 4B). A specific substitution at position 8 (^8^F → ^8^L) was found in the 19R44-3–2 sample belonging to lineage 5. In addition, amino acid mutations and higher amino acid entropy at position 10 in 50 PRRSV-2 and position 70 in 11 PRRSV-2 were found. Positive selection pressure analysis confirmed 18 codon sites in GP5 (Table 2). Four positive selection sites at positions 2, 5, 6, and 15 were included in the B cell epitope region (positions 1–15), two positive selection sites at positions 28 and 30 were included in the decoy epitope region (positions 27–30), and five positive selection sites at positions 32, 33, 34, 35, and 59 were included in hypervariable regions 1 and 2. Subsequently, two positive selection sites at positions 102 and 104 were included in the B cell epitope regions, and one positive selection site at position 151 was included in the T cell epitope region (positions 149–163). A total of four codon sites were found to be positively selected in M protein of PRRSV-2 (Table 2). One positive selection site at position 10 was included in the B cell epitope region, and three positive selection sites at positions 15, 16, and 66 were included in the T cell epitope regions (Table 2).

### Sensitivities and specificities of three one-step real-time RT-PCR assays

The sensitivities of three one-step real-time RT-PCR assays with two reference strains (Lelystad and LMY strain) were estimated to be 10^0^ TCID_50_/100μL. In the specificities of all one-step real-time RT-PCR assays determined by testing respiratory disease-causing viruses, such as PCV2, PCV3, CSFV, PPV, and SIV, no cross-reactivity was observed (data not shown).

### Clinical evaluation of three one-step real-time RT-PCR assays

Twenty PRRSV-negative clinical samples showed the same results in all one-step real-time RT-PCR assays. As shown in Table 3, the A test detected 100% of PRRSV-1 (47/47) and PRRSV-2 (62/62) in the samples. The B test detected 100% of PRRSV-1 (47/47) and 98.39% of PRRSV-2 (61/62) in the samples. Only 18D283-1 sample, belonging to Kor A of PRRSV-2, was not detected in the B test. When identifying inhibitors in the 18D283-1 sample, none of the diluted samples was detected (data not shown). Meanwhile, the C test detected 72.34% of PRRSV-1 (34/47) and 69.35% of PRRSV-2 (43/62) in the samples. There were 13 undetected samples of PRRSV-1 belonging to subgroups A (*n* = 12) and subgroup C (*n* = 1), and 19 undetected samples of PRRSV-2 belonged to Kor C (*n* = 8), Kor A (*n* = 4), lineage 1 (*n* = 4), Kor B (*n* = 2), and lineage 5 (*n* = 1). On comparison with the nucleotide sequences of the primers and probe in the C test, several nucleotide mismatches were observed in the forward primer sequences. The forward primer against PRRSV-1 was located at positions 14,792–14,809 in ORF7 of the Lelystad strain. Several mismatches were observed of nucleotide positions 14,792 (G/A), 14,795 (C/T), 14,798 (C/T), 14,801 (C/T), 14,804 (C/T), 14,805 (C/T), 14,806 (A/T), and 14,808 (A/G) (Supplementary Fig. 1A). The forward primer against PRRSV-2 was located at positions 15,257–15,274 in 3′ end of ORF7 and 3′-untranslated regions (UTR) of the VR2332 strain. Several mismatches were identified at nucleotide positions 15,257 (A/G/T), 15,261(T/C), 15,262(G/A), 15,263(G/A/T), 15,265(C/T/A), 15,267(G/A), 15,268(G/T/A), 15,269(C/T), 15,270(A/T) 15,271(T/C), 15,272(T/C), and 15,274(C/T). In particular, the 18R10-52–1 sample, belonging to Kor C of PRRSV-2, has 15 nucleotide deletions in the detection region of the forward primer (Supplementary Fig. 1B).

**Table 3:** Comparison of three one-step real-time RT-PCR with 47 PRRSV-1 and 62 PRRSV-2

Genotype	Number of samples	Number of positive (%)		
		A test	B test	C test
PRRSV-1	47	47 (100)	47 (100)	34 (72.34)
PRRSV-2	62	62 (100)	61 (98.39)	43 (69.35)
Total	109	109 (100)	108 (99.08)	77 (70.64)

## Discussion

After 30 years of PRRSV emergence, PRRSV infection remains a critical disease that causes enormous economic losses to the swine industry worldwide. Despite widespread efforts to control and prevent PRRSV infection, the virus has rapidly spread worldwide and has increasing genetic diversity [55]. The evolutionary rate of PRRSV (4.71–9.8 × 10^–2^/sites/year) is the highest among RNA viruses [56], which allows genetic diversity within PRRSV and the emergence of new phenotypes. In this study, a phylogenetic analysis of Korean PRRSV was performed using clinical samples collected from January 2018 to September 2021. Our results are consistent with previous findings in which all Korean PRRSV-1 belong to pan-European subtype 1, and most belong to subgroup A. On the other hand, Korean PRRSV-2 belongs to lineage 1, lineage 5, and Korean lineages (Kor A, B, and C) and the majority of Korean PRRSV-2 belongs to lineage 5 [13, 41]. However, in this study, lineage 1 shows the highest prevalence (25/62, 40.32%) followed by Kor C (13/62, 20.97%), Kor B (10/62, 16.13%), lineage 5 (9/62, 14.52%), and Kor A (5/62, 8.06%). A recent report showed that the PRRSV-2 lineage 1 population increased from 2014 (1.8%) to 2019 (29.6%) in Korea owing to the spread of sublineage 1.8 (NADC30-like viruses) and introduction of sublineage 1.6, comprising the second-largest population after lineage 5 (31.1%) in 2019 [41]. These changes may support the hypothesis for these epidemic situations such as the importing of breeding pigs and artificial insemination [57, 58]. Although the prevalence of PRRSV-2 in Korea should be confirmed in a larger sample size, we speculate that lineage 1 will become highly prevalent over time in Korea.

Previous studies showed ORF5 sequence homology of 85.8–90.9% between Korean PRRSV-1 and Lelystad virus in 2005–2009, and 84.9–98.4% in 2013–2016 [13]. The ORF6 of Korean PRRSV-1 showed sequence homology of 93.2–98.6% among the Korean strains and 85.6–94.4% among the non-Korean strains in 2012 [59]. The ORF7 of Korean PRRSV-1 showed a sequence homology of 88.8–99.7% among the Korean strains and 79.1–95.0% among the non-Korean strains in the 2000s [11, 14]. In this study, the lowest nucleotide sequence homology of ORF5 between PRRSV-1 and the Lelystad strain was 79.74%, indicating a decrease of 6.06% over approximately 15 years. The lowest homologies of ORF6 and ORF7 among the different PRRSV-1 isolates were 85.85% and 86.52%, respectively, indicating decreases of 7.35% and 2.28%, respectively. The ORF5 sequence homology between Korean PRRSV-2 and VR2332 was 84.7–99.5% in 2003–2010 and 82.3–99.3% in 2013–2016 [13]. The ORF6 nucleotide sequence identity among the Korean PRRSV-2 strains was 85.5–98.2% [60]. The ORF7 of Korean PRRSV-2 showed a sequence homology of 86.2–100.0% with each other and 88.3–100% with isolates from other geographic regions [61]. In this study, the lowest nucleotide sequence homology of ORF5 between PRRSV-2 and VR2332 was 78.71%, indicating a decrease of 5.99% in over approximately 20 years. The lowest homologies of ORF6 and ORF7 among the different PRRSV-2 isolates is 83.21% and 79.66%, respectively, indicating decreases of 2.29% and 6.54%, respectively. Previous study showed nucleotide substitution rates of 1.46 × 10^–3^ for PRRSV-2 viruses and 3.29 × 10^–3^ substitutions/site/year for two genotype isolates based on ORF5 sequences data [16, 62] and 4.17–9.8 × 10^–2^ substitutions/site/year based on ORFs 3–5 sequences of two genotype PRRSVs [56]. Consistent with theses previous investigations, our results also indicate that Korean PRRSV has high substitution rates of 5.8521–7.9748 × 10^–3^ for Korean PRRSV-1 and 4.516–5.7664 × 10^–3^ for Korean PRRSV-2. Therefore, it is suggested that the mutation rate of PRRSV circulating in Korea has increased over time.

In this study, the neutralizing epitope of PRRSV-1 was found to be conserved, but the GP5-III and M-I regions of PRRSV-1 were variable. Recent studies on the amino acid analysis of GP5 of Korean PRRSV-1 also showed a relatively conserved pattern in the B cell epitope regions, except for GP5-III (aa 165–176) [13, 41, 63]. GP5 and M of PRRSV-1 are not susceptible to antibody-mediated virus neutralization, in contrast to GP5 of PRRSV-2 is generally considered the main target for virus-neutralizing antibodies [50]. To understand the mechanism of neutralizing antibody against the Korean PRRSV-1, further analysis of the neutralizing antibody-escape mutants of PRRSV in other minor envelope glycoproteins such as GP2, GP3, and GP4 is required. The residue at position 44 of PRRSV-2 GP5 plays a critical role in virus infectivity; position 50 of GP5 and position 8 of the M protein are essential for assembly of PRRSV particles [64, 65]. In this study of PRRSV-2, the key residues at position 44 of GP5 and position 8 of M protein were variable in some samples. The key residues at positions 102 and 104 of PRRSV-2 GP5, which determine susceptibility to viral neutralization, were variable [66]. Residues (V102C and G104R) that were identical to those in a neutralizing antibody-escape mutant and with higher amino acid entropy were found in our several samples under positive selective pressure. In addition, amino acid mutations at positions 10 and 70 of PRRSV-2 M protein are related to susceptibility to viral neutralization [53, 54]; these features were validated in our samples. The hypervariable regions can modulate the accessibility of neutralizing antibodies to the neutralizing epitope [67]. Recently, variability in the decoy epitope region and hypervariable regions of GP5, as well as mutations in key residues related to neutralizing antibody-escape mutants, have been commonly found in Korean PRRSV-2 amino acid analysis studies [13, 41, 60]. These variations were also found among the PRRSV-2 samples in this study. Therefore, Korean PRRSV-2 has evolved to genetic variants with resistance to neutralization and may be able to escape neutralization by antibodies that are induced by commercial PRRS modified live vaccines (MLV).

A positive selection signal was detected in the neutralizing epitope region of PRRSV-1. Incidentally, sequence analysis of PRRSV-2 revealed variation and positive selection pressure within the decoy epitope and the neutralizing epitope regions. Recent research demonstrated that vaccination resulted in the emergence of antibody-escaping mutants, in which strong positive selection contributed to amino acid substitutions [68]. In a comparative analysis of PRRSV genetic diversity before and after vaccine adoption in Korea, PRRSV vaccination increased positively selected sites and the emergence of new glycosylation sites [63]. Currently, several commercial PRRS MLVs are available in Korea for the control of PRRSV infections. In this respect, it can be inferred that PRRSV vaccination leads to affecting positive selection, resulting in the emergence of escape variants.

Several reports have shown that genetic variability of PRRSV resulting in mutations at the primer binding sites leads to failure of RT-PCR tests [35, 69]. In the B test, only one sample (18D283-1) was not detected for PRRSV-2, which was not owing to an inhibitory effect. However, the nucleotide sequence could not be confirmed because the information on the primers and probe of the B test was confidential. The C test showed the lowest detection rate of 72.34% (34/47) for PRRSV-1 positive samples and 69.35% (43/62) for PRRSV-2 positive samples. On comparing the sequence of the false-negative samples of PRRSV-1 or PRRSV-2 and primers or probes of the C test, several nucleotide differences were found in several PRRSV-1 and PRRSV-2 positive samples. In general, inconsistencies in diagnostic results are known to be more common in the detection of PRRSV-1 than PRRSV-2. This observation could be explained by the large genetic diversity of the viruses within the PRRSV-1 genotype [35, 44, 70–72]. However, this study showed similar proportions of inconsistencies in the detection of PRRSV-1 and PRRSV-2 samples in the C test, suggesting that the genetic diversity of the viruses within PRRSV-2 was also increased.

## Conclusions

Our results demonstrate that two one-step real-time RT-PCR assays (A and B tests) efficiently detected PRRSV-1 and PRRSV-2 in the clinical samples. However, considering the emergence of the dominant population, diversification of evolution within the epitope regions of PRRSV structural protein, and characteristics of PRRSV in which genetic mutations continue to occur, frequent evaluation of diagnostic methods is essential for an accurate diagnosis of PRRSV. In addition, regular monitoring of the emergence of new PRRSV will provide information on the implementation of control and preventive measures against PRRSV.

## Supplementary Information

**Additional file 1: Figure 1.** Comparison between forward primer sequences of C testand target sequences of 13 PRRSV-1 samples (A) and 19 PRRSV-2 samples (B). Thematched nucleotide sequences between the forward primer and target sequenceswere hidden. Genome sequences of PRRSV false-negative samples were submitted toGenBank under accession numbers ON892746, ON892752, and ON892756–ON892781.

## Data Availability

The datasets supporting the conclusions of this article are included within the article. Some ORF5, ORF6, and ORF7 genome sequences among the all sequences obtained in this study were submitted to GenBank under accession number ON892744–ON892781.

## Abbreviations

PRRSV: Porcine reproductive and respiratory syndrome virus
ORF: Open reading frame
GP5: Glycoprotein 5
M: Membrane protein
RT-PCR: Reverse transcription polymerase chain reactions
OIE: World Organization for Animal Health
Kor: Korean lineages
MAbs: Monoclonal antibodies
SIV: Swine influenza virus
APQA: Animal and Plant Quarantine Agency
TCID_50_/ml: 50% Tissue culture infective dose per ml
PCV2 and PCV3: Porcine circovirus type 2 and type 3
CSFV: Classical swine fever virus
PPV: Porcine parvovirus
RFU: Relative fluorescence units
aa: Amino acid
MLV: Modified live vaccines
