# Supplementary material for: Genetic diversity of porcine reproductive and respiratory syndrome virus and evaluation of three one-step real-time RT-PCR assays in Korea
Source: BMC Vet Res. 2022 Aug 30;18:327. doi: 10.1186/s12917-022-03407-0 (PMC9429472; doi:10.1186/s12917-022-03407-0)
Supplement: Supplementary file 1 — Additional file 1: Figure 1. Comparison between forward primer sequences of C testand target sequences of 13 PRRSV-1 samples (A) and 19 PRRSV-2 samples (B). Thematched nucleotide sequences between the forward primer and target sequenceswere hidden. Genome sequences of PRRSV false-negative samples were submitted toGenBank under accession numbers ON892746, ON892752, and ON892756–ON892781. [file 12917_2022_3407_MOESM1_ESM.pdf]

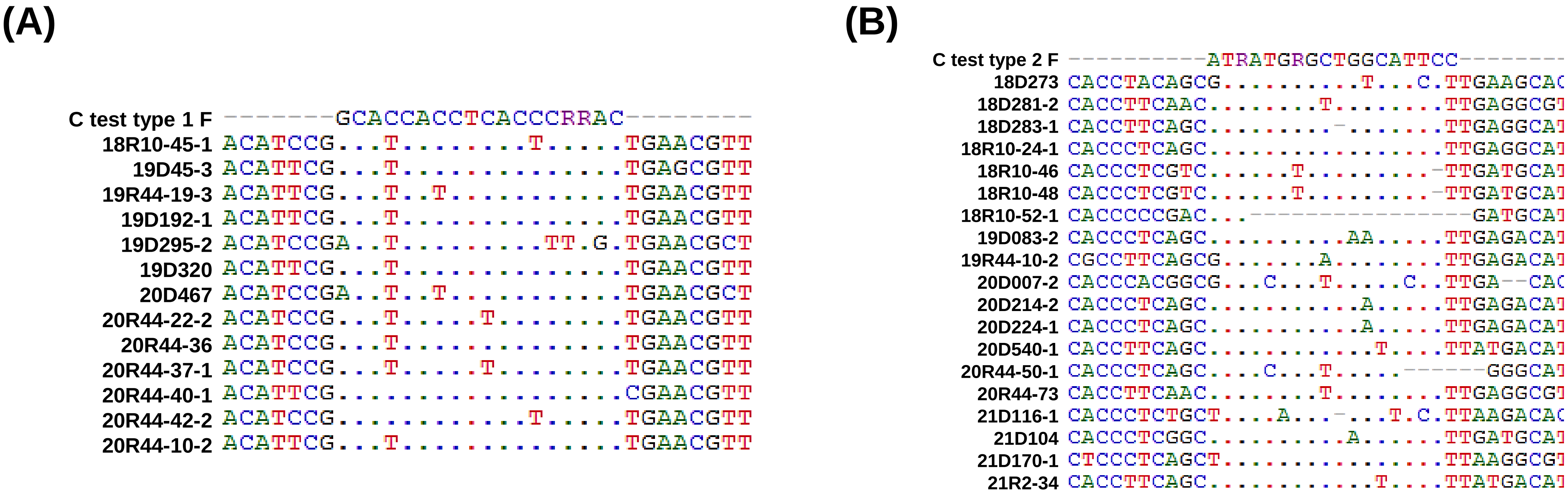

Supplementary figure 1. Comparison between forward primer sequences of C test and target sequences of 13 PRRSV-1 samples (A) and 19 PRRSV-2 samples (B). The matched nucleotide sequences between the forward primer and target sequences were hidden. Genome sequences of PRRSV false-negative samples were submitted to GenBank under accession numbers ON892746, ON892752, and ON892756–ON892781.
